# Immunomodulation Induced by Stem Cell Mobilization and Harvesting in Healthy Donors: Increased Systemic Osteopontin Levels after Treatment with Granulocyte Colony-Stimulating Factor

**DOI:** 10.3390/ijms17071158

**Published:** 2016-07-19

**Authors:** Guro Kristin Melve, Elisabeth Ersvaer, Çiğdem Akalın Akkök, Aymen Bushra Ahmed, Einar K. Kristoffersen, Tor Hervig, Øystein Bruserud

**Affiliations:** 1 Department of Immunology and Transfusion Medicine, Haukeland University Hospital, N-5021 Bergen, Norway; guro.kristin.melve@helse-bergen.no (G.K.M.); einar.kristoffersen@uib.no (E.K.K.); tor.audun.hervig@helse-bergen.no (T.H.); 2 Department of Clinical Science, University of Bergen, N-5020 Bergen, Norway; 3 Department of Biomedical Laboratory Sciences and Chemical Engineering, Faculty of Engineering and Business Administration, Bergen University College, N-5020 Bergen, Norway; elisabeth.ersver@hib.no; 4 Department of Immunology and Transfusion Medicine, Oslo University Hospital, Ullevål, N-0424 Oslo, Norway; uxciak@ous-hf.no; 5 Division for Hematology, Department of Medicine, Haukeland University Hospital, N-5021 Bergen, Norway; aymen.bushra.ahmed@helse-bergen.no

**Keywords:** allogeneic transplantation, hematopoietic stem cell mobilization, granulocyte colony-stimulating factor, osteopontin, apheresis

## Abstract

Peripheral blood stem cells from healthy donors mobilized by granulocyte colony-stimulating factor (G-CSF) and harvested by leukapheresis are commonly used for allogeneic stem cell transplantation. The frequency of severe graft versus host disease is similar for patients receiving peripheral blood and bone marrow allografts, even though the blood grafts contain more T cells, indicating mobilization-related immunoregulatory effects. The regulatory phosphoprotein osteopontin was quantified in plasma samples from healthy donors before G-CSF treatment, after four days of treatment immediately before and after leukapheresis, and 18–24 h after apheresis. Myeloma patients received chemotherapy, combined with G-CSF, for stem cell mobilization and plasma samples were prepared immediately before, immediately after, and 18–24 h after leukapheresis. G-CSF treatment of healthy stem cell donors increased plasma osteopontin levels, and a further increase was seen immediately after leukapheresis. The pre-apheresis levels were also increased in myeloma patients compared to healthy individuals. Finally, in vivo G-CSF exposure did not alter T cell expression of osteopontin ligand CD44, and in vitro osteopontin exposure induced only small increases in anti-CD3- and anti-CD28-stimulated T cell proliferation. G-CSF treatment, followed by leukapheresis, can increase systemic osteopontin levels, and this effect may contribute to the immunomodulatory effects of G-CSF treatment.

## 1. Introduction

Osteopontin is a glycosylated phosphoprotein synthesized and secreted by various cells [1]. The ability to interact with several cell surface receptors, including certain integrins and CD44, makes osteopontin a functional regulator of cell adhesion, migration, and survival for a wide range of cells [1]. Binding of osteopontin to the intracellular part of CD44 is important for cytoskeletal functions [2,3], transcriptional regulation, and anti-apoptotic signaling in normal and malignant cells [1,4,5,6]. Finally, osteopontin is important for normal hematopoiesis and is a component of the hematopoietic stem cell niche, where it regulates the location and cycling of normal stem cells [7,8].

Osteopontin is widely expressed by immunocompetent cells and upregulated both during inflammation and in various tumors [1,9,10,11,12,13,14,15]. It has pro-inflammatory effects by stimulating chemotaxis of various immunocompetent cells and by increasing pro-inflammatory cytokine release from macrophages [9] and expression of antigen-presenting and costimulatory molecules by dendritic cells [16]. It is also important for B cell proliferation and immunoglobulin production and is released by activated B cells and T cells as a Th1-associated cytokine [17,18,19]. However, osteopontin may also have anti-inflammatory effects [1], as observed both in animal models [19,20] and human disease [20,21].

Osteopontin is also important for growth regulation of acute lymphoblastic, and probably also acute myeloid leukemia, cells located at the endosteal stem cell niche [22,23]. Studies in humans have demonstrated that plasma osteopontin levels can reflect local inflammation [24] as well as tumor hypoxia and, thereby, chemo-sensitivity [25].

Systemic administration of granulocyte colony-stimulating factor (G-CSF) is commonly applied to mobilize hematopoietic stem cells for collection by leukapheresis [26,27,28]. Several apheresis systems have been developed for efficient harvesting of mononuclear cells [29,30,31]. Peripheral blood stem cell grafts are widely used for allogeneic and autologous hematopoietic stem cell transplantation (allo- and auto-HSCT) in hematological diseases, solid tumors and immune disorders [26,32,33,34,35,36], and increasingly in autoimmune and non-malignant gastrointestinal diseases [37,38,39]. Additionally, G-CSF mobilized progenitor cells are applicable in regenerative medicine and immunotherapy, and have, e.g., been tried in coronary and limb ischemia, as a possible source for differentiation of dendritic cells and for isolation of mesenchymal stromal cells [40,41,42,43,44].

One important complication associated with allo-HSCT is acute graft versus host disease (acute GVHD). The risk of acute GVHD seems to be similar for peripheral blood and bone marrow allografts [45], suggesting that the potentially adverse effect of the larger number of donor T cells in peripheral blood allografts is counteracted by immunomodulation of graft T cells during mobilization or harvesting.

Animal models suggest that osteopontin stimulates CD8^+^ T cell-mediated GVHD [46]. This effect may be caused either by pre-transplant modulation of immunocompetent cells in the allogeneic stem cell grafts, or by post-transplant modulation caused by osteopontin in the graft supernatant or osteopontin released in the recipient. Osteopontin has several immunomodulatory effects, and in this context we investigated the levels of osteopontin in autologous and allogeneic stem cell donors and stem cell grafts during mobilization/harvesting and in allogeneic stem cell recipients following graft infusion.

## 2. Results

### 2.1. Plasma Osteopontin Levels of Healthy Stem Cell Donors Increase during Granulocyte Colony-Stimulating Factor (G-CSF) Treatment and Reach a Maximal Level Immediately Following Stem Cell Harvesting by Leukapheresis

**Figure 1:**
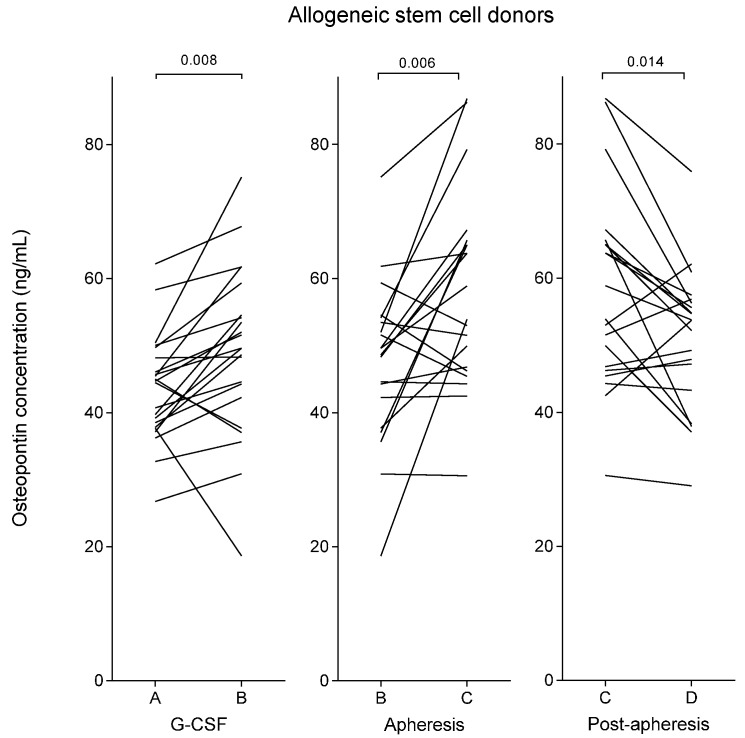
Plasma osteopontin levels in healthy allogeneic stem cell donors during stem cell mobilization and harvesting. Peripheral blood plasma osteopontin concentrations were determined prior to stimulation with granulocyte colony-stimulating factor (G-CSF) (A), after stem cell mobilization and immediately prior to apheresis (B), immediately after apheresis (C) and approximately 24 h after start of apheresis (D).

The median plasma osteopontin levels in healthy allogeneic stem cell donors prior to G-CSF therapy was 45 ng/mL (variation range: 27–62 ng/mL), see Table 1 and Figure 1. During G-CSF treatment, and immediately prior to leukapheresis, the osteopontin concentration in the stem cell donors was increased to a median level of 50 ng/mL (range: 19–75 ng/mL, *p* = 0.008). The healthy allogeneic stem cell donors were compared to a group of 15 healthy platelet donors who did not receive any kind of treatment prior to the apheresis. These healthy platelet donors showed no significant differences compared to the healthy stem cell donors with respect to age, gender distribution, or baseline white blood cell counts (Table 2). The pre-apheresis osteopontin concentrations of the platelet donors (median 44 ng/mL; range: 28–60 ng/mL) did not differ from the pre-treatment levels of the allogeneic stem cell donors either (Table 1).

The G-CSF-treated allogeneic stem cell donors showed a further increase of the median osteopontin concentration to 56 ng/mL (range: 31–87 ng/mL, *p* = 0.008, Table 1) immediately after leukapheresis, but 18–24 h after start of apheresis the median level had declined to 54 ng/mL (range: 29–76 ng/mL, *p* = 0.014, Figure 1). In contrast, the control group of healthy platelet donors showed stable osteopontin levels throughout the observation period without significant altered concentrations immediately after apheresis or 18–24 h after start of apheresis (Table 1).

Plasma G-CSF concentrations in allogeneic stem cell donors prior to and after mobilization were also investigated. The median pre-treatment G-CSF level was 50 pg/mL (range: 22–241 pg/mL) and after four days of G-CSF it was 10,780 pg/mL (range: 3687–31,947 pg/mL); see lower part of Table 1. G-CSF and osteopontin levels then showed no significant correlation.

There were no significant associations between osteopontin plasma levels and apheresis time (median: 305 min; range: 231–377 min) the absolute number of total blood volumes processed during apheresis (median: 3.6; range: 1.6–6.6), or apheresis device applied.

### 2.2. Plasma Osteopontin Levels Show an Inverse Correlation with Peripheral Blood Neutrophil Levels during G-CSF Therapy but No Association with Peripheral Blood Levels or Yields of CD34^+^ Cells

We used simple linear regression analyses with one way analysis of variance (ANOVA) to study the correlation between healthy stem cell donor osteopontin levels (all donors included in the analysis) and the corresponding peripheral blood levels of total leukocytes (Table 2) and leukocyte subsets. Plasma osteopontin levels immediately prior to leukapheresis showed significant inverse correlations with the corresponding peripheral blood neutrophil counts (median: 38.5 × 10^9^/L; range: 24.3–66.4 × 10^9^/L; *R*^2^ = 0.381; *p* = 0.002) and total peripheral blood leukocyte counts (median: 46.0 × 10^9^/L; range: 30.1−76.3 × 10^9^/L; *R*^2^ = 0.366; *p* = 0.003). With this exception, there were no significant associations between osteopontin levels and the total leukocyte counts or the levels of neutrophils, monocytes, total lymphocytes, CD3^+^ lymphocytes, or CD34^+^ cells in peripheral blood or in the stem cell graft at any other time point.

### 2.3. Myeloma Patients (Autologous Stem Cell Donors) Show Increased Plasma Osteopontin Levels after G-CSF Therapy Compared with Healthy Allogeneic Stem Cell Donors

**Figure 2:**
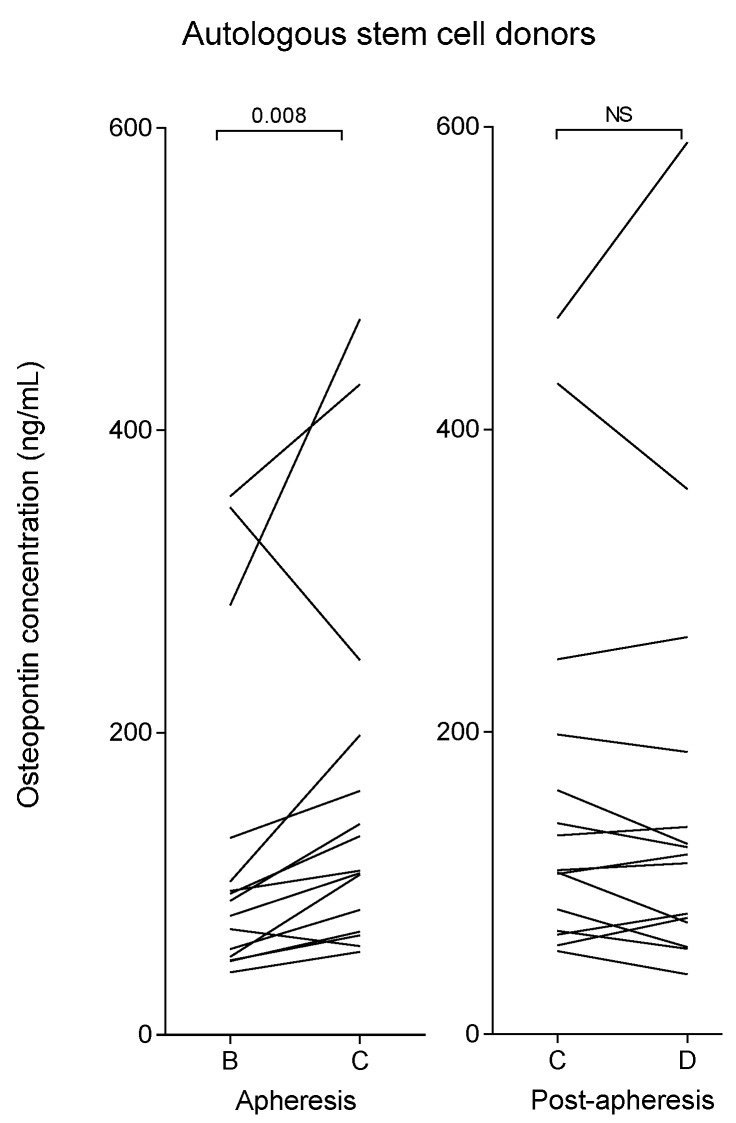
Plasma osteopontin levels in autologous stem cell donors (myeloma patients) after stem cell mobilization and immediately prior to apheresis (B), immediately after apheresis (C) and approximately 24 h after start of apheresis (D).

Plasma samples from myeloma patients receiving G-CSF therapy for mobilization of autologous stem cells were available only immediately before leukapheresis (after five days of G-CSF treatment); the plasma osteopontin levels then showed a wide variation and were significantly increased for the myeloma patients (median 89 ng/mL; range 41–356 ng/mL) compared with the pre-apheresis levels of the healthy stem cell donors (Mann-Whitney U test, *p* = 0.001). As presented in Table 1 (lower part), the pre-harvesting G-CSF levels were also significantly higher for myeloma patients (median 18,366 pg/mL; range 9861–46,314 pg/mL) than for the healthy stem cell donors (median: 10,780 pg/mL; range: 3687–31,947 pg/mL; *p* = 0.005). There was no significant correlation between pre-harvesting G-CSF and osteopontin plasma levels in the myeloma patients. As shown in Table 1 and Figure 2, myeloma patients had a significant increase in plasma osteopontin level during apheresis, but the increase in median osteopontin level 24 h after apheresis did not reach statistical significance.

### 2.4. Osteopontin Levels Are Higher in Autografts from Myeloma Patients than in Allografts from Healthy Stem Cell Donors

**Figure 3:**
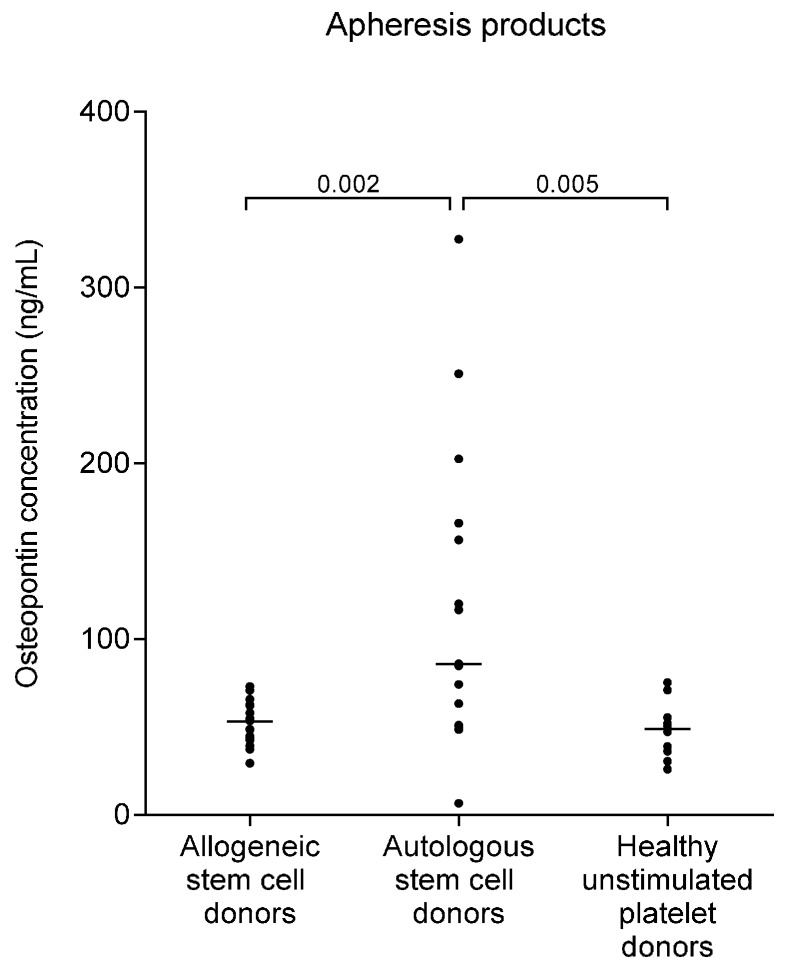
Osteopontin levels in apheresis products, i.e., peripheral blood stem cell grafts and platelet concentrates. The osteopontin levels were determined in allogeneic stem cell products from G-CSF-mobilized healthy stem cell donors (*n* = 22), autologous stem cell products derived from myeloma patients mobilized by chemotherapy plus G-CSF (*n* = 15), and platelet concentrates from unstimulated healthy platelet donors (*n* = 15). The osteopontin levels measured in platelet concentrate supernatants were adjusted for dilution of the products with platelet additive solution (37% plasma, 63% solution).

We then compared osteopontin concentrations in the apheresis products from autologous and allogeneic stem cell donors and healthy platelet donors. Autologous stem cell grafts from myeloma patients showed significantly higher supernatant osteopontin levels than the allografts (*p* = 0.002) and the platelet concentrates (*p* = 0.005); the results are summarized in Table 1 and presented in detail in Figure 3. The osteopontin levels in auto- and allografts were higher than unstimulated plasma levels in autologous and allogenic donors, but did not differ significantly from the corresponding plasma levels during G-CSF therapy. Due to dilution with platelet additive solution as described in the experimental section, the osteopontin levels in platelet concentrates were lower than the corresponding plasma levels in the platelet donors, and low compared to allogeneic and autologous stem cell grafts (median: 18 ng/mL; range: 10–28 ng/mL). The patients treated with the platelet concentrates thus received relatively low amounts of osteopontin during platelet infusion. However, after correction for the dilution factor, there was no significant difference between osteopontin levels in platelet concentrates and stem cell grafts from healthy donors or between platelet concentrates and peripheral blood samples from the platelet donors (Table 1, Figure 3).

### 2.5. Pretransplant Osteopontin Levels of Allotransplant Recipients Are Increased and the High Levels Are Not Altered Following the Infusion of Osteopontin-Containing Stem Cell Grafts

The pre-transplant osteopontin levels in allotransplant recipients were high (median: 126 ng/mL; range: 80–438 ng/mL) and were significantly higher than the levels in healthy individuals (*p* < 0.001; see Table 1), and even higher than for the myeloma patients (*p* = 0.02). The infusion of the osteopontin-containing allograft did not alter the plasma levels significantly; the levels remained high in the allotransplant recipients both when tested one day post-transplant and for eight patients also tested later after the transplantation (median: six days after infusion; range: 4–13 days).

Additional analyses showed no association between recipient osteopontin plasma levels (Table 1) and (i) patient age and gender; (Table 2) (ii) allograft content of leukocytes, CD34^+^ stem cells, CD3^+^ T cells, neutrophils, monocytes, lymphocytes or platelets measured as absolute numbers or as the number of cells per kg patient body weight (Table 3).

As presented in Table 3, the median time until neutrophil reconstitution with peripheral blood neutrophil counts above 0.5 × 10^9^/L on the first of three consecutive days was day +17 (range: day +13 to +28). Furthermore, the median time of platelet counts above 50 × 10^9^/L for the first of three consecutive days was day +15 (range: day +11 to +39). There was no significant association between osteopontin levels and time until hematopoietic reconstitution. Finally, for the 16 patients investigated acute GVHD grade II–IV was seen in two patients, early death before day +100 in four patients, chronic GVHD in nine patients, and leukemia relapse in four patients. These observations suggest that our 16 patients are representative for allotransplanted patients.

### 2.6. T and B Lymphocytes Show High Expression of the CD44 Osteopontin Receptor and these High Levels Are Maintained during Stem Cell Mobilization and Harvesting

**Figure 4:**
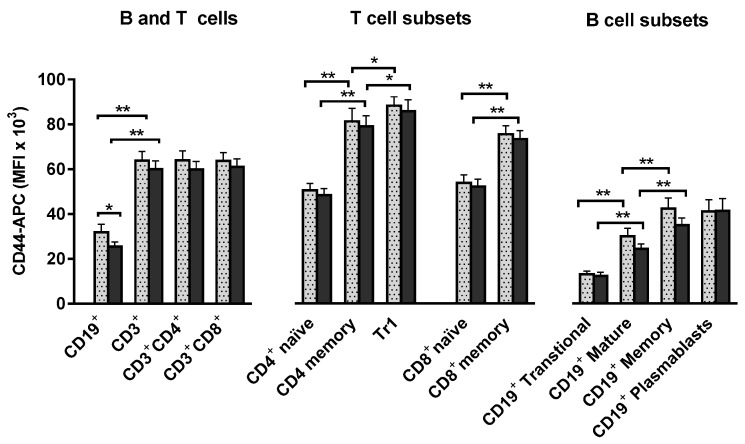
Expression of CD44 in unstimulated (grey-colored bars) and in vivo G-CSF stimulated (black-colored bars) peripheral blood leukocytes from healthy allogeneic stem cell donors. The results are presented as the mean fluorescence intensity (MFI) given as mean values ± standard error of the mean (SEM). (**Left**): The results for CD19^+^ B cells and CD3^+^ T cells with CD4^+^ and CD8^+^ main subsets are shown; (**Middle**): CD4^+^ and CD8^+^ naïve (CD45RA^+^) T cell subsets are compared with the corresponding T cell memory (CD45RA^−^) subsets and with T regulatory type 1 (Tr1) cells (CD4^+^ CD45RA^−^CD49b^+^ LAG-3^+^); (**Right**): Transitional B cells (CD19^+^CD24^hi^CD38^hi^) together with mature (CD19^+^CD24^+^CD38^+^) and memory (CD19^+^CD24^hi^38^−^) B-cells and plasmablasts (CD19^+^CD24^low^CD38^hi^) are presented. Statistically significant differences are indicated (** *p* = 0.001, * *p* = 0.05).

Interaction between osteopontin and the CD44 receptor mediates chemotaxis of lymphocytes and macrophages [47]. We investigated the expression of CD44 by viable donor lymphocytes during stem cell mobilization and harvesting; the receptor was generally highly expressed and all comparisons are therefore based on the mean fluorescence intensity (MFI), see Figure 4. In CD19^+^ B cells MFI was reduced from 31,869 to 25,519 (mean values, *n* = 15) during G-CSF stimulation (*p* = 0.022). No significant G-CSF induced change in CD44 expression was detected in CD3^+^ T cell populations; neither was there any significant effect of apheresis on CD44 expression in T and B cells. T cell and B cell CD44-APC MFI did not show any significant correlation to plasma levels of osteopontin or G-CSF at any sampling point.

CD44 expression was consistently higher for CD3^+^ T cells than for CD19^+^ B cells; as expected, both CD4^+^ and CD8^+^ CD45RA^−^ memory T cells showed significantly higher CD44 expression than CD45RA^+^ naïve T cells (Figure 4). Particularly high CD44 expression was found in the subset of CD49b^+^ LAG-3^+^ Tr1 cells (lymphocyte activation gene-3 positive T regulatory type 1 cells) [48].

We also compared CD44 expression in the main CD19^+^ B cell subsets [49], in unstimulated and G-CSF stimulated peripheral blood mononuclear cells (PBMC) samples. Compared to the CD24^+^CD38^+^ mature subset, transitional CD24^hi^CD38^hi^ cells showed significantly lower and CD24^hi^38^−^ memory B cells significantly higher CD44 expression. CD19^+^CD24^low^CD38^hi^ plasmablasts showed high CD44 expression similar to B memory cells [50].

To summarize, in vivo G-CSF therapy resulted in a modest reduction in CD44 expression in B cells exclusively, and apheresis procedures did not alter T and B cell CD44 expression significantly.

### 2.7. Osteopontin Causes a Minor Increase of in Vitro Proliferative T Cell Responses

**Figure 5:**
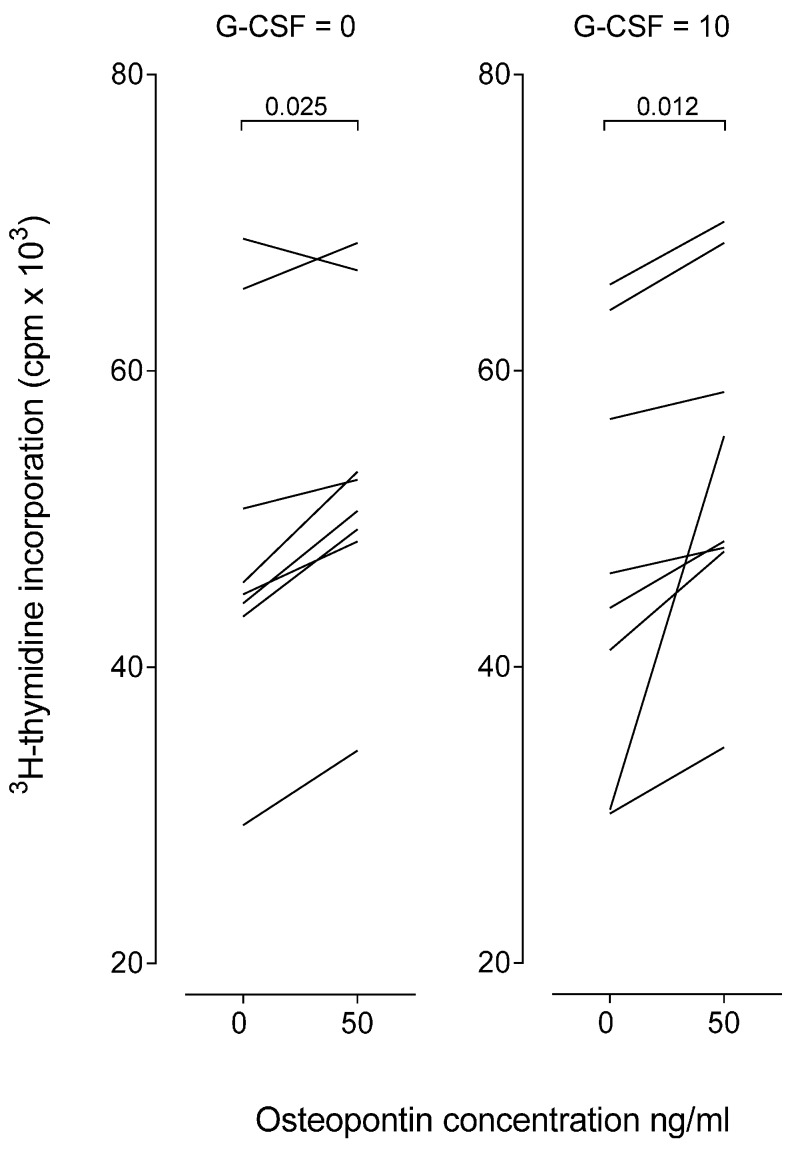
Peripheral blood mononuclear cells (PBMC) from eight healthy unstimulated donors were cultured in serum-free medium and stimulated with anti-CD3 and anti-CD28. The effect of osteopontin 50 ng/mL without G-CSF (**left**) and with G-CSF 10 pg/mL (**right**) on in vitro T cell proliferation was assayed as ^3^H-thymidine incorporation expressed as median counts per minute (cpm). The proliferation of normal PBMC in control cultures containing isotypic control antibodies instead of anti-CD3/anti-CD28 antibodies corresponded to <1000 cpm.

The effect of exogenous osteopontin on T cell proliferative responses was investigated for eight healthy individuals (Figure 5). PBMC were cultured in vitro in the presence of anti-CD3 and anti-CD28. We compared the proliferative responses for cultures prepared in medium alone and cultures with osteopontin 50 ng/mL, i.e., the osteopontin level corresponding to the plasma level in healthy stem cell donors (see Table 1). Osteopontin increased T cell proliferation, but this increase usually corresponded to less than 20% of the corresponding control cultures both when osteopontin was tested in culture medium without G-CSF and medium supplemented with G-CSF.

## 3. Discussion

Osteopontin can mediate both pro- and anti-inflammatory effects through its binding to specific receptors expressed by various immunocompetent cells [20,21]. In the present study we describe that systemic osteopontin levels are altered during stem cell mobilization and harvesting. Elevated osteopontin levels are detected in the stem cell grafts, and we hypothesize that osteopontin may thereby affect the immunocompetent cells in the grafts.

Some of the statistically significant differences in osteopontin plasma levels described in our present study were relatively small. However, the biological day-to-day variation, time of day variation, and week-to-week variation in osteopontin level in healthy blood donors has been shown to be low [51]. Furthermore, several previous studies have demonstrated that differences corresponding to 15%–25% of control levels reflect differences of biological and clinical significance, e.g., in cancer patients and cardiovascular disease patients [52,53,54]. These observations suggest that even relatively small variations in plasma osteopontin levels may have a clinical/biological relevance. Our own observations are also in agreement with these previous observations, e.g., we had similar results in base-line samples for our two independent groups of healthy individuals.

Our present study compared plasma osteopontin levels in two independent groups of healthy individuals (G-CSF treated stem cell donors, untreated platelet donors) undergoing apheresis with or without G-CSF stimulation. Osteopontin concentrations increased during G-CSF treatment, and the levels showed a further increase after leukapheresis/stem cell harvesting. This was a transient effect and osteopontin levels decreased during the 24 h period post harvesting. On the other hand, the control group of healthy untreated platelet donors showed stable osteopontin levels with no detectable effect of the apheresis.

We also compared the healthy allogeneic stem cell donors with a group of myeloma patients receiving G-CSF treatment for mobilization of autologous stem cells; the myeloma patients then showed higher pre-harvesting osteopontin levels and a similar increase as the healthy donors following leukapheresis. The higher pre-harvesting osteopontin concentrations in myeloma patients may be due to the combination of G-CSF and chemotherapy for autologous stem cell mobilization in these patients and five days of treatment with G-CSF in contrast to four days of treatment in the allogeneic donors. Alternatively, the difference could be disease dependent; increased levels in myeloma patients are associated with disease burden and decrease when patients respond to anti-myeloma treatment [55,56]. It should be emphasized that only a minority of our patients achieved a complete response prior to the autologous stem cell harvesting.

Samples drawn prior to G-CSF therapy were not available from our myeloma patients. In a recent study of myeloma patients mobilized for stem cell harvest, no significant effect of G-CSF on osteopontin levels could be detected [57]. However, as the regulation of the osteopontin concentration during stem cell mobilizing in these patients is complex and influenced by both disease stage and chemotherapy [55], possible effects of G-CSF might be difficult to detect.

Thus, the effect of apheresis (and possibly the effect of G-CSF treatment) on osteopontin levels is not only seen in healthy donors, but also in myeloma patients. However, the levels were not altered in healthy blood donors undergoing unstimulated thrombapheresis, which suggests that this is probably an effect induced by the G-CSF therapy and not a general effect of all kinds of apheresis procedures. This is further supported by reports of a relatively high degree of product manipulation and activation in the apheresis device used for platelet collection [58,59]. In contrast to our findings, an eventual effect of apheresis procedures on osteopontin levels would, therefore, be expected to be stronger during platelet collection compared to stem cell apheresis. However, it is not possible to exclude that differences in apheresis techniques between stem cell harvesting and platelet collection (e.g., processed blood volume, separation techniques, anti-coagulation) contributed to the different effects of apheresis on osteopontin levels.

G-CSF treatment both in healthy individuals and myeloma patients caused increased levels of circulating neutrophils that express the osteopontin receptor CD44 [60]. One would, therefore, expect increased binding of osteopontin to neutrophils during G-CSF treatment, but despite this increased binding we could still detect increased osteopontin plasma levels during the treatment.

A recent study of patients with hematological malignancies described an association between genetic CD44 polymorphisms and the efficiency of CD34^+^ cell mobilization [61], suggesting that CD44-osteopontin are important regulators of stem cell retention to the bone marrow during G-CSF mobilization, at least in myeloma patients. However, we did not observe any association between osteopontin levels and CD34^+^ cell mobilization/yield, neither in the myeloma patients, nor in the healthy stem cell donors.

We investigated the osteopontin levels in the graft supernatants. The high pre-harvesting plasma levels and the difference between healthy stem cell donors, myeloma patients, and platelet donors were also reflected in the osteopontin levels in the supernatants. The stem cell transplantation thereby also includes an infusion of osteopontin.

The osteopontin receptor CD44 is widely expressed by immunocompetent cells; the T cell expression was not altered by in vivo G-CSF exposure whereas B cell expression was moderately decreased. Exposure of T cells to osteopontin during in vitro activation caused a slight increase in anti-CD3 + anti-CD28 initiated T cell proliferation. These experiments show that osteopontin can alter T cell responses when tested at concentrations corresponding to the in vivo levels. However, additional studies are required to clarify whether this is a direct stimulatory effect on the proliferating cells, a reduced effect of T regulatory cells or an indirect effect mediated by the accessory cells.

The highest levels of osteopontin were found in allogeneic stem cell transplant recipients at the time of transplantation. The levels were high even compared to myeloma patients who had received both induction therapy and stem cell mobilization, and they were not significantly changed by stem cell transplantation. This observation indicates that high osteopontin concentrations is one of the characteristics of the pro-inflammatory state induced by conditioning therapy and underlying disease in allogeneic stem cell transplant recipients. This pro-inflammatory cytokine balance is considered as an important basis for development of GVHD [45], and osteopontin blockade is shown to reduce CD8^+^ T-cell mediated GVHD in mice [46]. Our findings suggest greater importance of the osteopontin level in the patient compared to the donor and stem cell graft. The osteopontin levels during conditioning therapy and allogeneic stem cell transplantation in humans and the possible importance for development of GVHD should be studied in further detail in order to evaluate osteopontin as a possible therapeutic target in graft versus host disease.

Previous studies have demonstrated that G-CSF has immunomodulatory effects and can suppress T lymphocytes [62].Such effects are probably important in allotransplant recipients receiving peripheral blood stem cell grafts because the frequency of GVHD is similar for bone marrow and mobilized peripheral blood stem cell grafts even though a higher frequency would be expected for the blood grafts due to their larger number of T cells in these grafts [62]. The molecular mechanisms behind this are not known, but our present study suggests that effects of osteopontin on immunocompetent cells may be a part of the G-CSF-induced immunomodulation in healthy stem cell donors. A better understanding of the mechanisms behind the G-CSF associated immunomodulation will be important for the future development of therapeutic strategies to target graft T cells and thereby reduce the risk of severe GVHD without reducing the graft versus leukemia reactivity.

## 4. Materials and Methods

### 4.1. Stem Cell Donors and Allotransplant Recipients

All studies were conducted in accordance with the Declaration of Helsinki and approved by the local ethics committee (REK III No. 126.01, Regional Committee for Medical and Health Research Ethics of Western Norway: 2008/1580, 2011/996, 2011/1237, 2011/1241, and 2013/634) and donors and patients were included after signing a written informed consent. The present studies included (i) 22 consecutive healthy human leukocyte antigen matched (HLA-matched), related, allogeneic stem cell donors; (ii) 15 consecutive autologous stem cell donors, all patients with newly-diagnosed symptomatic multiple myeloma; (iii) 16 allogeneic stem cell transplant recipients; and (iv) 15 healthy platelet donors (Table 2). The allogeneic stem cell donors did not differ from myeloma patients and healthy platelet donors with regard to age, gender distribution, or initial peripheral blood leukocyte count.

### 4.2. Stem Cell Mobilization in Healthy Donors and Myeloma Patients

The matched related donors received stem cell mobilizing with human non-glycosylated G-CSF 10 µg/kg per day for four days before stem cell harvesting. Initial induction therapy for the myeloma patients was two cycles of either intravenous cyclophosphamide 1 g/m^2^ on day 1 at four weeks intervals (14 patients) or bortezomib 1.3 mg/m^2^ on days 1, 4, 8, and 11 at a three-week interval (one patient); both regimens were combined with dexamethasone 40 mg orally on days 1–4 and 9–12. All myeloma patients either responded to the treatment or had stable disease, and stem cells were, thereafter, mobilized with intravenous cyclophosphamide 2 g/m^2^ followed by G-CSF 5 µg/kg/day. Peripheral blood leukocyte counts were significantly higher in healthy stem cell donors compared to myeloma patients immediately before stem cell harvesting (*p* < 0.001, Table 2), but the peripheral blood concentration of CD34^+^ cells did not differ significantly between groups.

### 4.3. Apheresis Procedures

Stem cell quantification was started on day 4 or 5 of G-CSF stimulation for stem cell donors and myeloma patients, respectively. For the myeloma patients this corresponded to day 10 after the start of cyclophosphamide. Stem cell harvest was performed when the stem cell count exceeded 15–20 × 10^3^/mL. Large-volume leukapheresis with four times processing of the total blood volume on a Cobe Spectra cell separator, version 7 (Cobe Laboratories, Gloucester, UK) was used for nine of the healthy stem cell donors and all the myeloma patients; the other 13 healthy stem cell donors were harvested with a Spectra Optia cell separator, version 9 (Terumo BCT Inc., Lakewood, CO, USA). The automated mononuclear cells (MNC) procedure was used in accordance with the instructions from the manufacturer. The yield of CD34^+^ cells per kg bodyweight obtained by apheresis and the white blood cell count in the apheresis product did not differ significantly between groups. Finally, single-donor platelet concentrates from unstimulated healthy volunteer donors were prepared with a Fenwal Amicus cell separator (Baxter Healthcare Corp., Deerfield, IL, USA) and leukocyte-reduction provided by elutriation. The platelets were suspended in 37% plasma and 63% platelet additive solution (T-sol, Baxter Healthcare Corp.) as described in detail previously [63,64].

### 4.4. Allogeneic Stem Cell Transplantation

Eleven of the 16 allotransplant recipients were diagnosed with acute myeloid leukemia (AML), three with acute B cell lymphoblastic leukemia (B-ALL), one with myelofibrosis and one with myelodysplastic syndrome (MDS). All leukemia patients were in complete hematological remission at the time of transplantation. The patients received (i) myeloablative conditioning with intravenous busulfan plus cyclophosphamide and mesna (14 patients); or (ii) reduced intensity conditioning with intravenous fludarabine plus busulfan (two patients). All patients were transplanted with G-CSF mobilized peripheral blood stem cell grafts derived from HLA-matched family donors and received graft versus host disease (GVHD) prophylaxis with cyclosporine A, plus methotrexate. Neutrophil reconstitution was defined as neutrophil counts exceeding 0.2/0.5 × 10^9^/L for at least three consecutive days, and platelet reconstitution as at least three consecutive days with stable platelet counts exceeding 20/50 × 10^9^/L.

### 4.5. Preparation of Plasma and Peripheral Blood Mononuclear Cells (PBMC)

#### 4.5.1. Blood Sampling

Venous blood samples from the allogeneic stem cell donors were collected (A) prior to G-CSF stimulation at the time of the pre-transplant evaluation (median 20.5 days before apheresis). For the three study groups undergoing apheresis, blood samples were also drawn (B) in the morning immediately before apheresis, (C) immediately after apheresis, and (D) approximately 24 h after start of apheresis. All venous blood samples from allotransplant recipients were collected between 07:00 and 09:00. Samples for plasma preparation were collected into Vacuette 9NC tubes and samples for cell preparation into acid-citrate-dextrose solution A (ACD-A) tubes with sodium citrate and acid-citrate-dextrose solution A as anticoagulants (Greiner Bio-One GmbH, Kremsmünster, Austria). Samples from stem cell allo- and autografts and platelet concentrates were transferred to plastic tubes without additives.

#### 4.5.2. Preparation of Plasma Samples

The blood samples were centrifuged at 2000× *g* (myeloma patients and platelet donors) or 1310× *g* (allotransplant recipients) for ten minutes at room temperature within 30 min of sampling. The supernatants were immediately transferred to plastic tubes, frozen, and stored at −70 °C until analyzed.

#### 4.5.3. Preparation of PBMC Samples

After isolation by density gradient separation (Lymphoprep, AXIS-SHIELD PoC AS, Oslo, Norway; specific density: 1.077 g/mL), PBMC were dissolved in RPMI 1640 medium supplemented with 2 mmol/L l-glutamine, penicillin 100 IE/mL, streptomycin 0.1 mg/mL (Sigma-Aldrich, St. Louis, MO, USA), and 20% fetal bovine serum (FBS, Biowest, Nuaillé, France). 10% dimethyl sulfoxide (DMSO, Sigma-Aldrich, St. Louis, MO, USA) was used as cryoprotectant, and the vials were stored in liquid nitrogen at −150 °C after gradual cooling to −80 °C in Mr. Frosty Freezing Container (Thermo Fisher Scientific, Waltham, MA, USA).

### 4.6. Analysis of Plasma Osteopontin and G-CSF Concentrations

Plasma osteopontin levels were determined by enzyme-linked immuno-sorbent assays (ELISA) (Quantikine ELISA Human Osteopontin (OPN) Immunoassay from R&D Systems, Minneapolis, MN, USA). Plasma G-CSF concentrations were determined by Luminex analyses (R&D Systems, Minneapolis, MN, USA). All samples were analyzed in duplicates, strictly according to the manufacturer’s instructions.

### 4.7. Flow Cytometry Analyses

PBMC were thawed in a 37 °C water bath, dissolved in supplemented RPMI 1640 medium, and incubated for one hour (37 °C, a humidified atmosphere of 5% CO_2_) before incubation with near-IR fluorescent reactive dye (LIVE/DEAD Fixable Dead Cell Stain Kits, Molecular Probes, Eugene, OR, USA) for 30 min to determine cell viability. After washing in phosphate-buffered saline (PBS) with 1% bovine serum albumin fraction V (BSA, Roche Diagnostics GmbH, Mannheim, Germany) the cells were incubated for 20 min with the following mouse anti-human monoclonal antibodies: CD3-PE-Cy7 (SK7), CD4-PerCP-Cy5.5 (RPA-T4), CD8-V500 (RPA-T8), CD19-PerCP-Cy5.5 (SJ25C1), CD45-RA-V450 (HI100), and CD24-PE-Cy7 (ML5) (all from Becton Dickinson Biosciences-BD Pharmingen, San Diego, CA, USA), rat CD44-Ax 488 (IM7) and mouse CD49b-FITC (P1E6-C5) (both from BioLegend, San Diego, CA, USA), mouse CD38-PB (HIT2; EXBIO, Prague, Czech Republic) and goat LAG-3-PE (FAB2319P; R&D Systems, Minneapolis, MN, USA). Eight-color flow cytometry analysis was performed using a FACS Canto II flow cytometer (Becton Dickinson Biosciences-Immunocytometry Systems; San Jose, CA, USA). Acquisition of 30,000 CD3^+^ T cells or 10,000 CD19^+^ B cells per sample was endeavored, and cytometer performance was monitored daily with Cytometer Setup and Tracking Beads (Becton Dickinson Biosciences-BD Pharmingen, San Diego, CA, USA). The data were analyzed with FlowJo software version X (FlowJo LLC, Ashland, OR, USA).

### 4.8. Analysis of T-Cell Proliferation by ^3^H-Thymidine Incorporation

PBMC were cultured in 96-well microtiter plates (5 × 10^4^ cells per well, 190 µL medium per well), the culture medium being X-vivo10^®^ with 100 µg/mL gentamycin (BioWhittaker, Walkersville, MA, USA). The T cells were activated by anti-CD3 (clone CLB-T3/4.E, 1XE, PeliCluster, Sanquin, Amsterdam, The Netherlands; final concentration 316 ng/mL) and anti-CD28 (clone: CLB-CD28/1, 15E8 PeliCluster; final concentration 842 ng/mL). The corresponding control antibodies were purchased from R&D Systems (Abingdon, UK). The medium was supplemented with recombinant human osteopontin 50 ng/mL (R&D Systems, Minneapolis, MN, USA) and eventually recombinant human G-CSF 10 pg/mL (PeproTech EC Ltd., Rocky Hill, NJ, USA). After three days of culture ^3^H-thymidine (280 kBq per well added in 20 µL of saline; TRA 310, Amersham International, Amersham, UK) was added and cultures harvested 18 h later. The median count per minute (cpm) of nuclear radioactivity for triplicate cultures was used for all calculations.

### 4.9. Statistical Analyses

The statistical analyses were performed by the standard computer software package IBM SPSS Statistics 22 (IBM Corporate, Armonk, NY, USA). The Wilcoxon’s test for paired samples was applied for analyses of paired observations, and the independent samples Mann-Whitney U test for comparison of groups. The covariance between different continuous variables was studied with simple linear regression analyses with one way analysis of variance (ANOVA).

## Figures and Tables

**Table 1:** The effect of granulocyte colony-stimulating factor (G-CSF) treatment, apheresis procedures and allogeneic stem cell transplantation on plasma osteopontin (OPN; **Upper part**) and G-CSF (**Lower part**) concentration. (**Upper part**) From the top, the plasma OPN levels are presented for the four study groups: (i) prior to and after G-CSF treatment of allogeneic stem cell donors; (ii) immediately before and after apheresis and in the apheresis product for each study group undergoing apheresis; and (iii) in allotransplanted patients 8–12 h prior to start of stem cell infusion and 12–16 h after infusion; (**Lower part**) Plasma G-CSF concentrations are given for allogeneic stem cell donors prior to and after G-CSF treatment and for autologous stem cell donors only after the G-CSF therapy. All concentrations are given as medians with variation ranges in parentheses.

Patients/Donors	Procedure	Pre-Procedure OPN (ng/mL)	Post-Procedure OPN (ng/mL)	*p* Value	Apheresis Product OPN (ng/mL)
Allogeneic stem cell donors	G-CSF stimulation	45 (27–62)	50 (19–75)	0.008	-
	Stem cell apheresis	50 (19–75)	56 (31–87)	0.006	53 (29–73)
Autologous stem cell donors	Stem cell apheresis	89 (41–356)	109 (55–473)	0.008	86 (7–328)
Healthy platelet donors	Platelet apheresis	44 (28–60)	46 (33–56)	NS	48 (25–75) ^1^
Allogeneic HSC recipients	Allogeneic stem cell transplantation	126 (80–438)	103 (72–260)	NS	Not applicable
**Patients/Donors**	**Procedure**	**Pre-Procedure G-CSF (pg/mL)**	**Post-Procedure G-CSF (pg/mL)**	***p* Value**	**Apheresis Product G-CSF (pg/mL)**
Allogeneic stem cell donors	G-CSF stimulation	50 (22–241)	10,780 (3687–31,947)	0.0003	6673 (1704–21,152)
Autologous stem cell donors	G-CSF stimulation	Not determined	18,366 (9861–46,314)	Not determined	12,906 (8863–41,139)

^1^ The osteopontin values measured in platelet concentrate supernatants were adjusted for dilution of the products with platelet additive solution (37% plasma, 63% T-sol). NS, not significant.

**Table 2:** Clinical and biological characteristics of healthy stem cell donors, autotransplanted myeloma patients, healthy platelet donors, and allotransplant recipients. Number of individuals, age, and gender (M: male, F: female) are presented for each study group. Median basal white blood cell counts (WBC × 10^9^/L) are given for the study groups undergoing apheresis. White blood cell counts and peripheral blood (PB) concentrations of CD34^+^ stem cells before start of apheresis and yield of CD34^+^ stem cells are given for G-CSF stimulated allogeneic and autologous donors (multiple myeloma patients). All values are presented as medians with the variation ranges given in parentheses.

Group	Age	Gender (M/F)	Total White Blood Cell Count in the Grafts		CD34^+^ Cells after G-CSF Treatment	
			Baseline Level (×10^9^/L)	After G-CSF (×10^9^/L)	PB Level (×10^3^/mL)	Yield (×10^6^/kg)
Allogeneic stem cell donors (*n* = 22)	51 (25–77)	14/8	5.9 (3.1–13.4)	46.0 (30.1–76.3)	44.1 (16.7–147.8)	5.4 (0.8–22.4)
Autologous stem cell donors (*n* = 15)	57 (44–67)	9/6	5.4 (2.5–9.0)	10.8 (2.7–43.7)	39.9 (9.7–175.0)	5.3 (1.1–27.9)
Platelet donors (*n* = 15)	47 (26–62)	8/7	6.0 (4.7–13.5)	-	-	-
Allogeneic HSCT recipients (*n* = 16)	47 (35–63)	7/9	-	-	-	-

HSCT, hematopoietic stem cell transplantation.

**Table 3:** Allogeneic stem cell grafts derived from healthy donors; the levels of various cells in the grafts and the post-transplant clinical course of the allotransplant recipients. The cell content of the stem cell grafts infused to 16 allotransplant recipients is presented as the absolute numbers in the graft (graft content) and as the infused cell doses per kg (infused cells).

Cell Type	Graft Content (×10^8^)	Infused Cells (×10^6^/kg)	Post-Transplant Course ^1^	
Total WBC	791 (342–2495)	109 (376–3054)	Neutrophil reconstitution	17 (13–28)
CD34^+^ stem cells	4.6 (2.4–6.7)	5.5 (3.3–6.8)	Platelet reconstitution	15 (11–39)
CD3^+^ T cells	278 (71–490)	39 (10–61)	aGVHD	2/16
Neutrophils	285 (112–1048)	45 (15–133)	cGVHD	9/16
Monocytes	127 (18–563)	16 (3–69)	Early death	4/16
Lymphocytes	346 (105–759)	50 (14–96)	Relapse	4/16
Platelets	7068 (3176–11,449)	9607 (3655–14,260)	-	-

^1^ Neutrophil and platelet reconstitution is given as the first of three consecutive days after the transplantation with neutrophil counts above 0.5 × 10^9^/L and platelet transfusion independence with platelet counts above 50 × 10^9^/L. aGVHD: acute graft versus host disease grade II–IV, cGVHD: chronic graft versus host disease, early death: defined as death before day +100 after transplantation, WBC: white blood cell count. All values are presented as medians with the variation ranges given in parentheses or as fractions of the total number of 16 patients.

## Abbreviations

G-CSF	Granulocyte colony-stimulating factor
CD	Cluster of differentiation
HLA	Human leukocyte antigen
GVHD	Graft versus host disease
OPN	Osteopontin
HSC	Hematopoietic stem cell
HSCT	Hematopoietic stem cell transplantation
MFI	Mean fluorescence intensity
PBMC	Peripheral blood mononuclear cells
LAG-3	Lymphocyte activation gene 3
Tr1 cells	T regulatory type 1 cells
^3^H	Tritiated hydrogen
MNC	Mononuclear cells
AML	Acute myeloid leukemia
B-ALL	B cell lymphoblastic leukemia
MDS	Myelodysplastic syndrome
ACD-A	Acid-citrate-dextrose solution A
FBS	Fetal Bovine Serum
DMSO	Dimethyl sulfoxide
Near-IR	Near-infrared
PBS	Phosphate-buffered saline
BSA	Bovine Serum Albumin
